# Testing an Electronic Patient-Reported Outcome Platform in the Context of Traumatic Brain Injury: PRiORiTy Usability Study

**DOI:** 10.2196/58128

**Published:** 2025-01-23

**Authors:** Christel McMullan, Grace Turner, Ameeta Retzer, Antonio Belli, Elin Haf Davies, Laura Nice, Luke Flavell, Jackie Flavell, Melanie Calvert

**Affiliations:** 1 Centre for Patient Reported Outcomes Research, Institute of Applied Health Research, University of Birmingham, Birmingham, United Kingdom; 2 Sport, Exercise and Rehabilitation Sciences, University of Birmingham, Birmingham, United Kingdom; 3 Institute of Inflammation and Ageing, University of Birmingham, Birmingham, United Kingdom; 4 Aparito Ltd, Wrexham, United Kingdom; 5 Centre for Conflict Wound Research, University of Birmingham, Birmingham, United Kingdom

**Keywords:** usability study, usability, patient reported outcome, PRO, electronic patient reported outcome, ePRO, traumatic brain injury, TBI, think aloud, cognitive interviews, early warning, early detection, mobile phone

## Abstract

**Background:**

Traumatic brain injury (TBI) is a significant public health issue and a leading cause of death and disability globally. Advances in clinical care have improved survival rates, leading to a growing population living with long-term effects of TBI, which can impact physical, cognitive, and emotional health. These effects often require continuous management and individualized care. Traditional paper-based assessments can be cumbersome, potentially impeding regular monitoring of patient-reported outcomes (PROs). Electronic PROs (ePROs) offer a promising alternative by enabling real-time symptom tracking, which can facilitate early identification of issues, support shared decision-making, and improve outcomes for patients with TBI.

**Objective:**

This study evaluates the usability of an ePRO platform—Atom5—for individuals with TBI. By analyzing how patients use the system to report their symptoms, the study aims to identify usability issues, assess user satisfaction, and determine the potential of Atom5 to support ongoing patient-centered care.

**Methods:**

Atom5 was customized to enable individuals with TBI to report their symptoms. Usability testing was conducted through one-on-one sessions with participants recruited from Headway UK—an organization supporting brain injury survivors. Each participant took part in cognitive interviews using with the “Think Aloud” method, encouraging them to verbalize their thoughts and experiences while using the platform. This approach provided qualitative insights into areas of difficulty, usability strengths, and accessibility barriers. User satisfaction was quantitatively assessed with a brief 4-item questionnaire based on the System Usability Scale. Usability outcomes were analyzed for critical and noncritical errors, focusing on user experience and overall satisfaction.

**Results:**

In total, 9 participants completed a single usability testing session using Atom5, including 4 men, 4 women, and 1 nonbinary individual; 4 participants were under 55 years old, and 6 had their TBI <10 years ago. Finally, 8 participants used an Android device. The platform included measures for anxiety (Generalized Anxiety Disorder-2 item), depression (Patient Health Questionnaire-2), posttraumatic stress disorder (Posttraumatic Stress Disorder checklist 2), and TBI-specific quality of life (Traumatic Brain Injury – Quality of Life Short form) and a total of 26 questions. Overall, all participants were satisfied with the system, noting that it was easy to navigate and accessible despite difficulties in understanding some questions. Further, 6 participants encountered no errors, while 1 participant reported one critical error and 2 others reported one noncritical error each. The participants rated their overall satisfaction with the platform at an average score of 3.9 (SD 0.49) out of 5.

**Conclusions:**

This usability study suggests that individuals living with TBI can effectively report symptoms using the Atom5 ePRO platform, with generally high satisfaction and few usability issues, thereby enabling continuous monitoring and proactive symptom management. Future ePRO development should focus on inclusivity and adaptability to address the diverse needs of patients with TBI, ensuring these tools can effectively support a wide range of users.

## Introduction

### Background

A traumatic brain injury (TBI) is defined as “a traumatically induced structural injury and/or physiological disruption of brain function as a result of an external force” [1]. Each year, it is estimated that over 50 million TBIs occur worldwide [2]. In the United Kingdom, around 900,000 people attend accident and emergency departments (immediate critical care) with a head injury every year, with approximately 1.3 million people living with the effects of a TBI [3].

Although most TBIs result in mild symptoms and do not require hospitalization, many individuals experience disabling long-term symptoms [4-7]. The impact of a TBI falls into several categories: physical (headaches, dizziness, and blurred vision), cognitive (trouble with memory, concentration and attention, and impaired reasoning), emotional (personality changes, mood swings, depression, and anxiety), and behavioral (fatigue and anger) [8-11]. This means that patients with mild TBI have different needs, and health care must be tailored to each patient [12].

In addition, advances in management and guidelines [13] mean that people now live longer with the effects caused by their TBI. It is therefore important for clinicians to monitor these effects over time to improve the quality of life of people living with the effects of a TBI.

As a result, TBI is being increasingly regarded as a chronic condition, as it impacts on multiple health domains and functions [4,14]. Research has shown that TBI is a risk factor for dementia [14], psychiatric, cardiovascular, genitourinary and neurodegenerative conditions [15-17].

One way to monitor the effects of TBI on patients’ symptoms and quality of life over time is by using patient-reported outcomes measures (PROMs). PROMs are defined as “questionnaires completed by patients to assess the effects of disease or treatment (or both) on symptoms, functioning, and health related quality of life from their perspective” [18]. PROMs have traditionally been administered in a paper format. Research has shown that there are several drawbacks to paper PROMs. These include lengthy completion time, risk of poor data quality, and being prone to human error [19]. The growth of eHealth technologies and the omnipresence of smartphones and tablets [20] has allowed patients to play a more active role in their health care [21-23].

Digital health is a rapidly evolving field and provides a cost-effective way to remotely monitor patients [12,23]. Often referred to as mobile health or “mhealth,” recent research has shown that the use of these new technologies in health care has been received positively both by patients [12,24] and clinicians [24-26]. In addition to this, new technologies have given clinicians and patients the opportunity to use electronic PROMs (ePROMs) [20]. Unlike paper PROMs, ePROMs can facilitate real-time data, remote monitoring of symptoms and send or receive feedback [27], and can improve acceptance rate [28]. Examples of successful ePROs systems include AmbuFlex in Denmark with patients with renal failure [29] and the eRAPID system in the United Kingdom to monitor side effects of chemotherapy [30]. However, disadvantages of ePROMs include concerns around privacy issues, technical difficulties, a large initial financial investment, and the potential digital divide [19].

Like any new product being developed, it is vital to test the usability of a new electronic patient-reported outcome (ePRO) system in order to improve and optimize the final product [31,32]. Usability testing is defined by the International Organisation for Standardization (ISO) as “the extent to which a product can be used by specified user to achieve specified goals with effectiveness, efficiency, and satisfaction in a specified context of use” [33].

### Research Context: The PRiORiTy Study

This usability study is the second stage of the wider National Institute for Health and Care Research Surgical Reconstruction and Microbiology Research Centre (NIHR SRMRC)–funded PRiORiTy (Patient-Reported Outcomes Research in Trauma) study. The PRiORiTy study aimed to develop and test the usability and feasibility of an ePRO system with people with a TBI. Prior to this usability study, we conducted a qualitative study on the views and perspectives of patients living with the effects of a TBI, their carers or family members, and health care professionals on using PROMs or ePROMs. The findings of the qualitative study were reported in an article published elsewhere [27].

### Aim

The aim of this study was to test the usability of using an ePRO system (Atom5) with individuals who had a TBI.

## Methods

### Study Participants and Settings

People who had a TBI were recruited in July 2021 from Headway UK, a UK-based charity aiming to promote understanding of all aspects of brain injury and provide information, support and services to survivors, their families and carers. The sample size was informed by previous usability studies, which were in favor of the 10±2 rules, which advocated that around 10 participants could identify 80% of the issues around the system being tested [34-36]. Other studies showed that as low as 5 people are needed if conducting a series of testing sessions [31,37]. However, since we conducted only one testing session, we decided to increase our sample size and it was clear that we reached data saturation by the time we conducted our last testing session. Inclusion criteria were being 18 years of age and older, having the ability to converse in English, and to give informed consent. We excluded anyone who was not able to give informed consent.

### Recruitment Process

A lead contact within Headway UK identified people who were deemed eligible (purposive sampling), approached them in person, and introduced them to the study. The Headway UK staff member briefly explained the study to eligible people and gave them the information sheet. They then sent the researcher (CM), via a secure email address, the contact details of the people who had verbally agreed to take part in the usability study. CM phoned these people to arrange a mutually suitable date and time to conduct the testing session. Written informed consent was obtained from all the participants and data were anonymized.

### ePRO Platform (Atom5)

The ePRO platform used in this study was Atom5, which was developed by Aparito Ltd [38], a UK-based medical technology company. Atom5 consists of two interfaces (Multimedia Appendix 1):

1. A clinician dashboard accessed via a web browser that can be accessed by specific members of the research team with appropriate permission.
2. A patient-facing interface accessed via an app on Android or iOS devices onto which study participants input their patient-reported outcome data.

The Atom5 app for the PRiORiTy study was designed in conjunction with our Patient and Public Involvement and Engagement group, the research team (including a consultant neurosurgeon) and was informed by the findings from the project’s qualitative study [27].

Aparito Ltd are accredited for ISO 13485:2016 for medical device quality management system, ISO 27001:2001 for information security management systems, and Cyber Essential Plus recertification.

### PROMs Selection

The selection of PROMs was informed by the findings of our previous qualitative research [27], literature searches, and input from our patient partners and clinical collaborators. Our qualitative research showed that people who had a TBI often experienced anxiety, depression, and posttraumatic stress disorder, and this was further evidenced by the existing literature [8,9,27]. Therefore, the PROMs included in this study were the Generalized Anxiety Disorder-2 item (GAD-2), Patient Health Questionnaire-9 (PHQ-9), Posttraumatic Stress Disorder checklist 2 (PCL-2) and Traumatic Brain Injury – Quality of Life Short form (TBI QOL SF; ability to participate in social roles and satisfaction with social roles and activities). Quality of Life after Brain Injury (QOLIBRI) was considered as a potential TBI-specific measure. However, because it includes 37 items, the Patient and Public Involvement and Engagement and clinical collaborators advised against it, as they felt participants with TBI would not be able to complete it due to their inability to stay focused for long periods of time. Instead they suggested using 2 submeasures of the TBI QOL SF (ability to participate in social roles and satisfaction with social roles and activities) for a total of 20 questions. In addition, clinical collaborators had already been using TBI QOL questionnaire in their clinical practice.

The details of the four PROMs selected are mentioned in Table 1.

**Table 1.** Patient-reported outcome measures included in the Patient-Reported Outcomes Research in Trauma usability study.

PROM^a^ category and PROMs		Description
General population		
	GAD-2^b^ (Anxiety)	Screening tool for generalized anxiety disorder [39] derived from GAD-7^c^ Two items Recall period of 2 weeks Validated measure which has retained the same psychometrics properties of the GAD-7 (86% sensitivity and 83% specificity) [40]
	PHQ-2^d^ (Depression)	Screening tool for depression derived from the PHQ-9^e^ Two items Recall period of 2 weeks Validated measure with sensitivity of 83% and specificity of 92% [41]
	PCL-2^f^ (PTSD^g^)	Abbreviated version of the PTSD Checklist – Civilian version (PCL-C) Two items Screening tool for PTSD Recall period of 1 month Validated measure with sensitivity of .97 and specificity of .58 [42]
Disease-specific		
	TBI QOL SF^h^ (ability to participate in social roles; satisfaction with social roles and activities)	Health-related Quality of Life 20 items Recall period of 7 days Validated measures with excellent psychometric characteristics, although more research is needed to establish evidence of construct validity and evaluate the measures’ sensitivity to change [43]. In addition, the measures need to be validated when using via mobile health.

a PROM: patient-reported outcome measures.

b GAD-2: Generalized Anxiety Disorder-2 item.

c GAD-7: Generalized Anxiety Disorder-7 item.

d PHQ-2: Patient Health Questionnaire-2.

e PHQ-9: Patient Health Questionnaire-9.

f PCL-2: Posttraumatic Stress Disorder checklist 2.

g PTSD: posttraumatic stress disorder.

h TBI QOL SF: Traumatic Brain Injury – Quality of Life Short form.

### Data Collection and Testing Procedure

#### Testing Sessions

The usability testing aimed at assessing the effectiveness and efficiency of the ePRO platform. This consisted of one-to-one sessions conducted either online on a University of Birmingham secure Zoom account or face-to-face on Headway premises in the West Midlands. The testing sessions and interviews were conducted by CM, PhD, who is a female qualitative research fellow with extensive experience in qualitative data collection, including conducting interviews. She did not know the participants prior to the start of the research. The participants were fully informed of the reasons for doing the research prior to the data collection.

At the start of the session, participants were given a patient leaflet that outlined the main stages involved in navigating through the app and completing the questionnaires. They were then asked to download the app and perform a series of tasks on the app while sharing their thoughts about the platform (Think Aloud technique) [44]. Finally, the participants took part in a short interview (10‐15 min) on their experience of using the electronic platform. A carer or family member was allowed to attend the testing session. A topic guide was developed and pilot-tested with a patient with TBI. Field notes were kept during the testing sessions.

The tasks performed by the participants are detailed in Textbox 1.

Textbox 1. **Tasks performed by participants during the testing sessions.**

- Download the Atom5 app
- Onboard
- Consent
- Navigate through homepage
- Navigate through Questionnaires module
- Complete and submit Generalized Anxiety Disorder-2
- Complete and submit Patient Health Questionnaire-2
- Complete and submit Posttraumatic Stress Disorder checklist 2
- Complete and submit Traumatic Brain Injury – Quality of Life Short form
- Navigate through frequently asked questions module

#### Satisfaction Questionnaire

Finally, the participants were asked to answer a brief 4-question satisfaction questionnaire to rate their satisfaction with the usability of the electronic platform (5-point scale: 1–poor or never to 5–excellent or yes) [36,45].

#### Data Saturation

There has been an ongoing debate about sample sizes for usability testing. Our sample size has been been informed by previous research, which shows that 8‐10 participants are required to detect over 80% of issues.

### Data Analysis

Data were analyzed by two independent researchers. The participant characteristics were summarized as frequency and percentage. The testing sessions were audio-recorded. The participants’ comments during the testing sessions and the researcher’s observations were collated into a table. Errors were recorded and divided into critical (requiring the researcher’s input to be able to continue) and noncritical (not requiring the researcher’s input to be able to continue). Suggestions to improve the electronic platform were also recorded. Participant ratings for the 4 satisfaction questions were used to calculate a mean score per question. Overall mean score and SD was also calculated.

Data collected from the ePROMs was used for the purpose of the usability study only and was not downloaded.

The COREQ (Consolidated Criteria for Reporting Qualitative Research) reporting checklist was used to report findings (Checklist 1).

### Patient and Public Involvement and Engagement

One person who was living with the effects of a TBI and a family member were involved throughout the study. We met regularly (on average every 3 mo) and they reviewed patient-facing documents (patient information sheet, consent form, and instructions leaflet), assisted with recruitment and provided advice on the design and development of the Atom5 platform. In addition to meeting regularly, we were in regular email contact in order to quickly address any urgent issues.

### Ethical Considerations

The study was approved the University of Birmingham Ethics committee (reference ERN_17‐1253). Informed consent was obtained from all participants. The participants were not compensated for taking part in the study. All data were deidentified to ensure participant privacy and confidentiality.

## Results

### Participants’ Characteristics

A total of 9 people who had a TBI took part in a testing session. This included 4 men, 4 women, and 1 person identifying as nonbinary. A total of 4 out of the 9 participants (44%) were under 55 years old, and 5 were over 55 years old. The majority of the participants (6/9 participants, 66%) had their TBI less than 10 years ago. Finally, 8 out of 9 participants (89%) used an Android device, and 1 used an iOS device. The participants’ characteristics are summarized in Table 2.

In total, 5 testing sessions took place remotely by video using Zoom and the other 4 took place face-to-face on Headway UK premises. No one else was present in the interview room, although Headway UK staff were in the building. The testing sessions lasted between 42 and 63 minutes.

Among the 4 male participants, 3 of them were visually impaired and needed some help to read the questions. The researcher (CM) read the questions for them verbatim and helped them to input their answers onto the electronic device. These 3 male participants did not own an electronic device and had to borrow one from friends.

**Table 2.** Participants’ characteristics.

Variables		Values, n (%)
Age (years)		
	<55	4 (44)
	>55	5 (56)
Sex		
	Male	4 (44)
	Female	4 (44)
	Nonbinary	1 (12)
Ethnicity		
	White	9 (100)
Number of years since traumatic brain injury		
	<10 years ago	6 (67)
	10‐20 years ago	2 (22)
	>20 years ago	1 (11)
Device used		
	iOS	1 (11)
	Android	8 (89)

### Effectiveness and Efficiency of the Atom5 App

One critical error and 2 noncritical errors were recorded and all the participants completed the testing sessions. The critical error was due the participant not knowing what to do after inputting the onboarding code (please note that after downloading the Atom5 platform, users need to either scan a QR code or enter a numerical code to be able to access the questionnaires. The QR code or numerical code was given to them by the researcher (CM). The two noncritical errors were due to downloading the app on an iPad not compatible with the platform and not being able to answer a question because the question was missing. Table 3 shows how each participant addressed the critical and noncritical errors.

**Table 3.** Descriptions of critical and noncritical errors encountered by participants and how they were addressed during testing sessions.No differences were found between iOS and Android users.

Error (error type)	How participants addressed noncritical error
Not knowing what to do after inputting the onboarding code (critical error)	Researcher advised the participant
Downloading the app on an iPad not compatible with the platform (noncritical error)	Participant used a newer device
Not being able to answer a question because the question was missing (noncritical error)	Participant had to go back to previous page to read the question

It was difficult to record how long it took participants to complete the PROMs on Atom5 as they discussed their experience with the researcher while carrying out the tasks required. However, the researcher observed that while the majority had very little difficulty completing GAD-2, PHQ-2, and PCL-2 and completed them in under 30 seconds, most of the participants struggled with the TBI QOL SF questionnaire and took longer to complete it. One of the reasons for this is that it is a longer questionnaire (20 questions for TBI QOL SF vs 6 questions for GAD-2, PHQ-2, and PCL-2 combined). However, another reason for struggling with it was the difficulty understanding some of the questions, such as the following questions, and not knowing how to answer them:

- “I am able to do all the community activities that I want to do”
- “I can do everything for work that I want to do (including work at home).”
- “I am satisfied with the amount of time I spend doing work (including work at home).”

### Issues and Suggestions for Improvement

The participants identified a number of issues and made some suggestions to improve their experience of using the Atom5 platform (Table 4). These issues and suggestions related to font size, lack of clarity, names of questionnaires, questions numbers, clarity of some the text, what to do after onboarding, communication with clinical team, length of questionnaires, questions wording, colors, and name of the app.

**Table 4.** Summary of issues and suggestions for improvement made by participants during the testing sessions.

Issue	Details	Suggestions made by participants
Font size too small	Some items were difficult to read: Questions in GAD-2^a^ were difficult to read FAQs^b^	Increase font
Lack of clarity	Difficulty reading and understanding: Consent text FAQs Thank you message after questionnaires Unsure what the FAQs are about	Reformat using bullet points or add paragraphs Include information in FAQs about study and what participants have to do
Lack of information about study	What participants need to do after onboarding was not always obvious to all participants	Add instructions on app
Communication with clinical team	Need for some participants to leave comments	Add free text boxes at the end of each questionnaire
Length of questionnaires	TBI QOL SF^c^ was lengthy	Add the question number at the start of TBI QOL SF question (eg, 1/20; 2/20, etc)
Questions wording	Difficulty understanding some of the TBI QOL SF questions	Reword the questions^d^ Add emojis/pictures
Orange and white colors	Most participants did not understand the significance of the orange and white colors The white color was difficult to see by one participant	Add explanation in FAQs Increase font size Bold words that are on white background
Name of the app Atom5	Name of app was not related to TBI^e^	Change the name to make it more relevant to TBI

a GAD-2: Generalized Anxiety Disorder-2 item.

b FAQ: frequently asked questions.

c TBI QOL SF: Traumatic Brain Injury – Quality of Life Short form.

d This may not be possible, as it may impact on validity of measures and would require work with the developers.

e TBI: traumatic brain injury.

### Participants’ Overall Comments and Researchers’ Observations

Overall, participants were positive about using Atom5 to report their symptoms. They found the app easy to download and use. Most of them liked the white and orange colors of the app, even though they did not understand the significance of it. The main issues mentioned by the participants was regarding the TBI QOL SF questions, which they had difficulty understanding and therefore were not sure how to answer them. One participant reported they would be unlikely to use the platform again because it would be too difficult to use on their own as they would forget to use it (Textbox 2).

Textbox 2. **Participants’ overall comments on using the Atom5 platform and researchers’ observations.**

**Participants’ comments**

- Very easy to use.
- App is quite good and looks smart.
- Easier than a piece of paper.
- Things are laid out very well in the app.
- Unlikely to use it again because it would be difficult to use on my own.
- I wouldn’t remember to use it.
- Downloading the app might be an issue for some people with a traumatic brain injury.
- Made sense but had to think about the questions.

**Researchers’ observations**

- Several participants had to read the Traumatic Brain Injury – Quality of Life Short form (TBI QOL SF) questions several times, difficulty understanding what the questions mean.
- Two participants were getting tired halfway through completing the TBI QOL SF questionnaire.

### Participants’ Satisfaction

The summary scores for participants’ satisfaction and rating of usability of Atom5 are shown in Table 5. The overall mean score was 3.9 (SD 0.33) and the mean score for individual questions was between 3.4 (SD 0.52) and 4.6 (SD 0.72).

**Table 5.** Participants’ usability and satisfaction scores of using the Atom5 electronic platform (range: 1‐5).

Question	Average score (SD)
Q1. How easy was the system to use and navigate?	3.9 (0.33)
Q2. How satisfied are you with the content?	4.6 (0.72)
Q3. How satisfied are you with the display?	3.4 (0.52)
Q4. How likely are you to use it again or recommend it to others?	3.9 (1.16)
Average usability and satisfaction score	3.9 (0.49)

## Discussion

### Summary of Findings

This article reports the results of the usability testing of the Atom5 platform used by people living with the effects of a TBI. Our ePRO platform was informed by existing literature, patients, and a clinician. A total of 4 PRO measures were selected and programmed in Atom5. The results of the interviews show that most of the people living with the effects of a TBI who took part in the usability study were able to report their symptoms using the Atom5 platform. The number of errors were low (1 critical and 2 noncritical errors) and were due to incompatibility of a device, not knowing what to do after onboarding, or a missing question. Overall, the platform was found to be effective. Suggestions to improve the electronic platform included increasing the font, reformatting the text and layout to make it clearer, and adding instructions or study information and free text boxes.

### Findings in Relation to Existing Literature

The results of our usability study reflect results from previous usability studies to some extent [24]. Although our participants were positive about their overall experience of using Atom5, their satisfaction scores were slightly lower than in other studies [36,45,46], reminding us that barriers to the use of ePRO platforms still exist. Participants’ lower satisfaction scores could be attributed to several factors, including visual difficulty, fatigue, cognitive impairment, and some questions difficult to understand and answer.

As we were aware that a TBI, even a mild TBI, can cause fatigue and attention deficit, among many other symptoms [47,48], we deliberately kept the number of questions as low as possible while being useful enough for the clinical team. However, a few participants started to feel tired a few minutes into the testing sessions. This is sometimes known as “respondent fatigue,” and it refers to the way respondents lose concentration as they complete questionnaires, especially in the latter part of the questionnaires [49]. The fact that all the participants managed to complete the questionnaires and the tasks required is encouraging and suggests that they believed in the potential of the ePRO platform enough to complete the testing sessions [50].

In addition, several participants had to read some of the questions several times, especially questions from the TBI QOL SF questionnaire. Even after reading the questions several times, they did not always understand them. While this could be due to a lack of concentration, it is also worth wondering if the wording of the questionnaires is suitable for individuals living with the effects of a TBI and whether the measure is suitable for use in routine care. As this issue might not directly relate to the ePRO platform, it would be important to explore this further in a future study.

One of the main risks of using ePRO platforms is excluding certain groups of patients [19,51]. This concerned 2 specific groups in our study. First, 3 participants who were visually impaired after their TBI had to be helped with reading the questions. Visual impairment is a common consequence of TBI [52], and therefore it is essential to include individuals who have visual impairment after a TBI. The second group of people who risks of being excluded is people who do not own an electronic device or are not familiar with technology [19]. A total of 3 of our participants did not have a smartphone or tablet and borrowed someone else’s device in order to take part in the usability study. This could, however, raise issues around data privacy and security.

Finally, 1 participant felt they would not remember to use the electronic platform unless they were sent a reminder and suggested being sent a reminder, which has been shown to increase response rate [53].

### Implications for ePRO Developers, Health Care Professionals, and Researchers

The fact that some of our participants were visually impaired should not exclude them from using ePROMs, as demonstrated in our study. In the case of our participants with visual impairment, the researcher read the questions. Therefore, ePRO developers, health care providers, and researchers should ensure electronic platforms are accessible for all, to ensure inclusivity and maximize participation [46] by allowing family members or carers to complete proxy measures [54] or by providing a “Read aloud” option for patients.

Similarly, although this was not the case with our participants, the suggestion that some people who had a TBI might struggle with downloading the app suggests issues around digital literacy. ePRO developers and researchers should ensure participants receive adequate training or support. The fact that 3 of our participants did not have access to a mobile device indicates that electronic devices should be provided to patients to allow them to complete ePROMs. This would also remove privacy and security issues if using their own devices or borrowing someone else’s. Maximizing participation could also be increased if ePRO developers sent reminders to participants.

Several participants mentioned they would have liked to see their previous answers to the questionnaires in order to view their progress over time. Therefore, adding visual representations of users’ previous answers would help them to do so.

Finally, it is recommended for health care professionals and researchers to use validated PROMs.

### Strengths and Limitations

The main strength of our study is the Think Aloud technique used for data collection; allowing participants to share their views while carrying out the requested tasks provides real-time feedback [44,55], in particular, about the questions from the TBI QOL that they had difficulty understanding and responding to.

There are several limitations to our study. The first one is the lack of ethnic diversity among participants, meaning that study findings might not be representative of the wider population. The research team recognizes the need to promote inclusive data collection. In addition, the lack of text-to-speech or voice commands in the app made it more difficult for participants with vision impairment to use the app.

The time it took participants to complete the tasks was not representative of the time it would take to complete the ePROs, as they shared their views while carrying out the tasks. This made it difficult to assess efficiency of the electronic platform.

The fact that, despite excellent psychometric characteristics and a reading age of fifth-grade level (10/11 years old), some of the participants struggled to understand and answer some of the questions, implying that their answers did not reflect how they felt.

Finally, the thinking aloud approach used in this usability study means that some of the participants could become a little tired during the testing sessions because of the cognitive issues some of them faced. Researchers should ask participants to reflect on their experience after each task rather than at the end of the session.

### Conclusion

Usability testing is a vital stage of any new product development. In our case, it showed that although individuals living with the effects of a TBI can report their symptoms on an electronic platform, ePRO developers, health care providers, and researchers should ensure that electronic platforms are inclusive and can be adapted to people’s needs.

Future research should include testing the Atom5 platform in TBI clinic settings and with ethnic minorities.

## Supplementary material

10.2196/58128 Multimedia Appendix 1 Screenshots of Atom5 app and clinical dashboard.

10.2196/58128 Checklist 1 COREQ (Consolidated Criteria for Reporting Qualitative Research) checklist.

## Abbreviations

COREQ: Consolidated Criteria for Reporting Qualitative Research
ePRO: electronic patient-reported outcomes
ePROM: electronic patient-reported outcome measures
GAD-2: Generalized Anxiety Disorder-2 measure
ISO: International Organisation for Standardization
mTBI: mild traumatic brain injury
NIHR SRMRC: National Institute for Health and Care Research Surgical Reconstruction and Microbiology Research Centre
PCL-2: Posttraumatic Stress Disorder Checklist 2
PHQ-2: Patient Health Questionnaire-2
PRiORiTy: Patient-Reported Outcomes Research in Trauma
PRO: patient-reported outcomes
PROM: patient-reported outcome measure
QOLIBRI: Quality of Life after Brain Injury
TBI: traumatic brain injury
TBI QOL SF: Traumatic Brain Injury – Quality of Life Short form
