# Supplementary material for: Testing an Electronic Patient-Reported Outcome Platform in the Context of Traumatic Brain Injury: PRiORiTy Usability Study
Source: JMIR Form Res. 2025 Jan 23;9:e58128. doi: 10.2196/58128 (PMC11781241; doi:10.2196/58128)

Appendix 1 – Screenshots of Atom5^TM^ and clinical dashboard used by the clinical staff


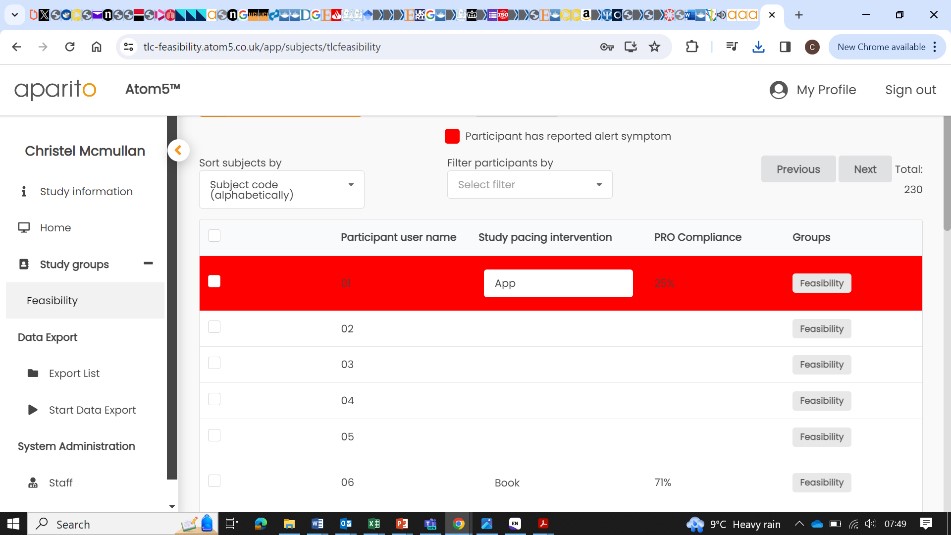


Appendix 2 – Screenshots of Atom5^TM^ patient-facing app used by the patient participants


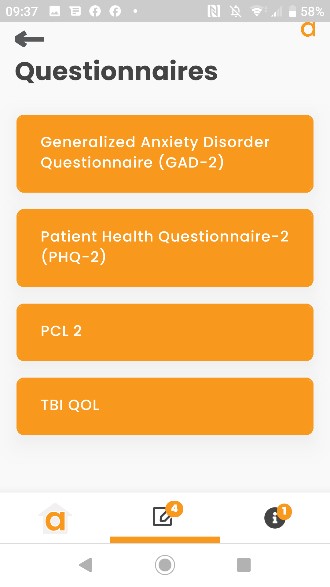

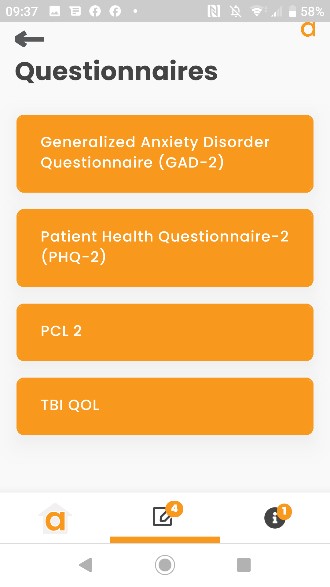

Supplement: Multimedia Appendix 1 [file formative-v9-e58128-s001.docx]
